# Dimension-Reduced Analog—Digital Mixed Measurement Method of Inductive Proximity Sensor

**DOI:** 10.3390/s17071533

**Published:** 2017-06-30

**Authors:** Yi-Xin Guo, Zhi-Biao Shao, Hui-Bin Tao, Kai-Liang Xu, Ting Li

**Affiliations:** 1 The School of Electronic and Information Engineering, Xi’an Jiaotong University, No.28, Xianning West Road, Xi’an 710049, China; zbshao@mail.xjtu.edu.cn (Z.-B.S.); agike@sina.com (T.L.); 2 The Institute of AI & Robotics, Xi’an Jiaotong University, No.28, Xianning West Road, Xi’an 710049, China; coldfire_arm@126.com; 3 The School of Automation and Information Engineering, Xi’an University of Technology, No.5 South Jinhua Road, Xi’an 710048, China; klxu@xaut.edu.cn

**Keywords:** inductive proximity sensor, displacement measurement, dimension-reduced method, characteristic of response curve, linear approximation method, look-up table

## Abstract

Inductive proximity sensors (IPSs) present a unique no-contact advantage. They are widely preferred for displacement measurement in various industrial fields (e.g., aviation and aerospace), and they are improved continuously. When the inductance and resistance components of the IPS sensing core are separated, the influence of temperature drift on measurement can be eliminated. The complexity of online computation of component separation can be reduced using a two-dimensional look-up table method. However, this method exhibits disadvantages, such as large capacity of the look-up table, dependency on precision measurement of sensing core parameter, and nonlinear distribution of measurement resolution. This study aims to overcome these disadvantages by examining the nonlinear relationship between the response of the sensing core and the ambient temperature, and proposes a dimension-reduced measurement method. The proposed method extracts the characteristics of the response curves at two temperatures and calculates the characteristics of the response curves at any temperature using a linear approximation. The look-up table capacity is less than 0.37% of the two-dimensional look-up table capacity (condensed) under the same condition; dimension reduction enables the construction of a complete look-up table directly by calibration procedures and avoids precise measurement on sensing core parameters; the calibration procedures establish uniform mapping of the distribution of measurement resolution. The experiment shows that, when the measurement ranges are 0–6, 0–5, and 0–4 mm, the maximum measurement errors are 0.140, 0.065, and 0.040 mm, respectively, under temperature ranging from 20 ${}^{\circ }$C to 110 ${}^{\circ }$C. This study extends the measurement range from 0–5 mm to 0–7 mm and improves the measurement accuracy over 0.1 mm (50% at 5 mm) compared with the two-dimensional look-up table method. Therefore, the proposed method not only inherits the advantages of the original method but also achieves the above-mentioned expected capacity improvements effectively.

## 1. Introduction

Detecting the position of moving metal objects is important in modern industrial production processes. Inductive proximity sensors (IPSs) meet the requirements of no mechanical contact, resistance to fouling and abrasion, water tightness [1], long service life, and low mechanical system maintenance cost [1,2]. High mean time between failure (MTBF) value [3], strong magnetic immunity [4,5,6], and built-in self-test (BIST) function are convenient for application [7,8,9]. IPS is applicable in the high-reliability industrial field of position detection, especially in aviation and aerospace [7,10,11].

For an aircraft, the required mechanical distance measurements are usually in the range of 2–6 mm, and the required measurements accuracy are in the range of 0.2–1.0 mm [7,10,12,13]. According to RTCA-DO-160F (a standard for the environmental testing of avionics hardware named environmental conditions and test procedures for airborne equipment), the required operating temperature ranges from −55–0 ${}^{\circ }$C and 55–70 ${}^{\circ }$C [10,12,13,14]. The sensors are located on the landing gear, doors, cargo loading systems, and thrust reverser actuation systems (TRAS) [10,11,13,15]. Traditional eddy-current-position switches can also measure the distance in this scope. However, these sensors use rapid variation in magnetic reluctance of cores as switching outputs, which seriously affects ambient electromagnetic environment [7,13,15,16]. In order to satisfy the emission of radio frequency energy testing of RTCA-DO-160F, any open magnetic circuit with large current excitation should be avoided in avionic applications [10,12,16]. Hence, IPS with small current excitation (usually less than 15 mA) is widely used in the field of aviation [12,13,15,16]. For example, the aircrafts Airbus (Blagnac, France) A380, A330, A320, Boeing (Seattle, WA, USA) B767, B747, and GE (Evendale, OH, USA) CF6-50 (TRAS) use special IPSs of Crane (Burbank, CA, USA) (80-008, 8-552, 72A04, 8-527, 8-527, 8-644) [10,16].

The MTBF values of complicated electromechanical system decrease exponentially with the enhancement in the system scale [3,17,18,19]. This enhancement of the system scale increases the demand for single-component stability [5,17,19]. For a single product, application effect difference between using micro-controller unit (MCU) and other simple circuit is not obvious. However, there is great stability difference for complicated electromechanical system containing dozens or hundreds of IPSs [12,15,16]. Hence, IPSs used in aviation usually avoid using process control components, such as MCUs and digital signal processors (DSPs).

The function principle of IPS is based on the interaction between the internal sensing coil and the external metallic target. The electromagnetic field established by the sensing coil is affected by the external target; thus, the inductance component of the sensing coil correlates to the distance between the IPS and the target [2,12,15,16,20]. However, as the sensing coil is formed by copper wire winding, its resistance component significantly changes with the ambient temperature [21,22]. Consequently, the accuracy of IPS measurement is largely constrained [23,24]. Traditional IPSs can detect the nearness of the target but cannot create quantitative outputs [25,26]. Reducing the disturbance of the temperature drift is the key point to improve the precision of the IPS system [12,27].

Many methods have been proposed, including analog [10,28,29] and digital measurement methods [30,31], to reduce the temperature drift of IPSs. Analog measurement procedures involve applying pulse excitation on a sensing coil and comparing the thresholds of the r-L response waveform with a comparator to determine the inductance value. A thermistor is commonly used to reduce temperature drift [32]. However, measurement precision is usually low because it is constrained by the consistency of the manufacturing process [33,34]. Meanwhile, as the working point of the analog system is affected by passive components, the stability of the analogy circuit is unstable in the long run [12,30,35]. Digital measurement procedures involve applying a sine wave excitation to a sensing coil, sampling voltage and current waveforms, using the Fourier transform algorithm to identify the phase difference between voltage and current, and calculating the inductance and resistance components of the sensing coil [21,30]. Separating the inductance and resistance components reduces temperature drift [12,30]. However, the digital signal processing of digital measurement method is complicated; thus, this process requires the use of a MCU or a DSP. This application negatively affects the miniaturization of IPS and is constrained in high-reliability industrial fields, such as aviation [12,15,16,36].

A new method of temperature-compensated inductance-to-frequency converter has been proposed by Matko and Milanović recently [37]. The influences of the temperature drift are strongly reduced by an oscillator switching method. This method can be introduced into the IPS measurement on the basis of further study on the miniaturization.

An analog–digital mixed measurement method has been proposed [12]; this method applies a pulse excitation on the sensing coil and takes two samples of the response waveform at specific moments. The inductance and resistance components are separated, and the measurement errors caused by temperature drift are reduced. The inductance component is deduced by seeking a two-dimensional look-up table. This method simplifies the online calculation and disregards the use of MCU and DSP. However, the method possesses the following disadvantages:

1. The look-up table is on a large scale (the look-up table can be condensed into approximately 50 k points when using a 12-bit analog-to-digital converter (ADC) [12]) and requires large capacity non-volatile memory, thereby negatively affecting the miniaturization of IPS [38,39,40].
2. The method depends on the precision measurement of IPS sensing coil parameter. IPS production requires accuracy of every product parameter and produces a special two-dimensional look-up table via offline computation, thereby negatively affecting scale production [41].
3. The two-dimensional look-up table is recorded in the form of inductive uniform variation. However, the inductance component of the sensing coil expands rapidly in nonlinear form when the distance between the IPS and the target reduces. This process leads to the non-uniform distribution of the measurement resolution [42].

This study modifies the disadvantages of the two-dimensional look-up table method by proposing a new dimension-reduced measurement method based on the nonlinear optimization of the correlation between the ambient temperature and the response waveform. This method only needs to extract the characteristics of the response curves at two temperatures and to calculate the characteristic of the response curve at operating temperature using a linear approximation. Therefore, this method avoids simultaneously traversing the ambient temperature and the distance in establishing the look-up table. This method also reduces the look-up table from two dimensions to one dimension; thus, the look-up table capacity is greatly decreased (less than 0.37% of the condensed one of two dimensions under the same condition [12]). This method helps control the scale of the look-up table during the process of further improving IPS measurement precision by increasing the resolution bits of ADC. Dimension reduction enables the use of precise electronically controlled displacement platform to directly calibrate the relationship between the distance and the response. Using the uniform growth of the distance results in better storage utilization efficiency to record the look-up table than using the uniform growth of the inductance. The calibrating process offsets the inconsistency of IPS sensing coil production and avoids precise measurement on the sensing core; this feature contributes to IPS scale production. On the basis of the analysis above, this study designs and develops IPS prototypes and a precise electronically controlled displacement platform. The experimental data shows that, when the measurement ranges are 0–6, 0–5, and 0–4 mm, the maximum measurement errors are 0.140, 0.065, and 0.040 mm, respectively, under the temperature ranging from 20 ${}^{\circ }$C to 110 ${}^{\circ }$C. This method is compared with that in the previous work based on the two-dimensional look-up table method under the same condition (by reducing the ADC resolution of this method from 14-bit to 12-bit). The distributions of the maximum measurement errors in the two studies are similar. Furthermore, the reduction in the look-up table dimensions of this method improves the sampling resolution from 12-bit to 14-bit with less memory consumption; in accordance with the simplification of the calibration procedures, the measurement range is extended from 0–5 mm to 0–7 mm, and the measurement accuracy is improved over 0.1 mm (50% at 5 mm). Therefore, the proposed method not only achieves the expected capacity improvements but also improves the measurement precision and extends the measurement distance.

## 2. Modeling of the Sensor Characteristics

**Figure 1:**
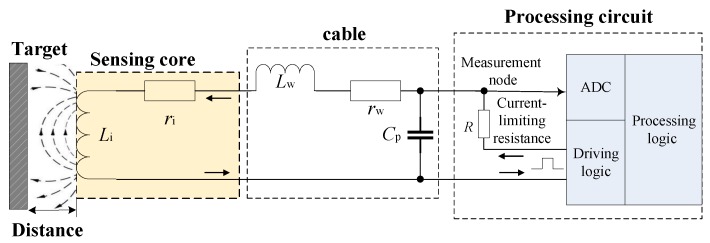
Block diagram of inductive proximity sensor.

As shown in Figure 1, IPS is composed mainly of the sensing core and the processing circuit. The sensing coil is equivalent to a series circuit of inductance component $L_{i}$ and resistance component $r_{i}$. $L_{i}$ shows the distance between the sensing core and the target; $r_{i}$ is affected by the ambient temperature of IPS. By driving and testing on the sensing coil, the processing circuit calculates $L_{i}$ and exports the distance between the sensing core and the target. For integrative IPS, the sensing core and the processing circuit are connected by a short wire and are installed into a case; for non-integrative IPS, a multi-channel processing circuit and several sensing cores are used in combination, and the sensing core and the processing circuit are connected by a cable (less than 10 m in general). $C_{p}$ is the equivalent distributed capacitance of the cable; $L_{w}$ is the equivalent distributed inductance of the cable, which is significantly less than $L_{i}$; $r_{w}$ is the internal resistance of the cable, which is significantly less than $r_{i}$.

The driving logic of the processing circuit creates driving waveform periodically and forms a charging circuit through the sensing core. Inductance component $L_{i}$ charges slowly through resistance component $r_{i}$, the cable, and current-limiting resistance *R*. The processing logic takes samples of the response waveform of the sensing core via ADC, and calculates the distance between the sensing core and the target.

**Figure 2:**
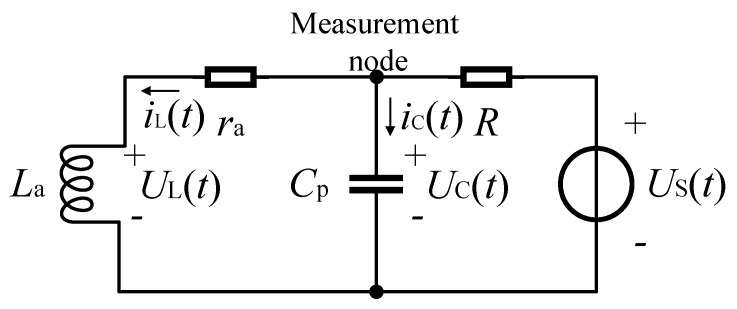
Equivalent circuit of the inductive proximity sensor (IPS).

Figure 2 shows the equivalent circuit of IPS. $L_{a}$ is equal to $L_{i}$ plus $L_{w}$, $r_{a}$ is equal to $r_{i}$ plus $r_{w}$, and $U_{S}\left(t\right)$ is the function of the driving waveform. $U_{C}\left(t\right)$ is a zero-state response on the measurement node of the second-order inertial system.

Using the rising edge of the driving waveform at the starting point, the model above can be described by a second-order differential equation. On the basis of the loop of $U_{S}$+, *R*, $r_{a}$, $L_{a}$, and $U_{S}$-, and the loop of $C_{p}$+, $r_{a}$, $L_{a}$, and $C_{p}$-, two independent Kirchhoff’s voltage law (KVL) equations are given by

(1) $$\left\{\begin{array}{ccc} R\left(i_{C}+i_{L}\right)+U_{C} & = & U_{S},\\ -U_{C}+r_{a}i_{L}+U_{L} & = & 0. \end{array}\;\right.$$

Substituting the voltage current relations into Equation (1), the differential equations of the equivalent circuit are

(2) $$\left\{\begin{array}{ccc} R\left(C_{p}\frac{dU_{C}}{dt}+i_{L}\right)+U_{C} & = & U_{S},\\ -U_{C}+r_{a}i_{L}+L_{a}\frac{di_{L}}{dt} & = & 0. \end{array}\;\right.$$

$i_{L}$ in the equation above should be eliminated to obtain the differential equation with the voltage on the measurement node $U_{C}$ as the variable. Taking the differential operator D=d/dt into Equation (2), following Cramer’s rule, the solution found above is

(3) $$RC_{p}L_{a}D^{2}U_{C}+\left(RC_{p}r_{a}+L_{a}\right)DU_{C}+\left(R+r_{a}\right)U_{C}=L_{a}DU_{S}+r_{a}U_{S}.\;$$

By applying inverse transform of the differential operator, the standard form of the differential equation with the voltage on the measurement node as the variable can be written as follows:

(4) $$RC_{p}L_{a}\frac{d^{2}U_{C}}{dt^{2}}+\left(RC_{p}r_{a}+L_{a}\right)\frac{dU_{C}}{dt}+\left(R+r_{a}\right)U_{C}=L_{a}\frac{dU_{S}}{dt}+r_{a}U_{S}.\;$$

Considering that $U_{S}\left(t\right)$ is a step input, the solution found of Equation (1) is

(5) $$\left\{\begin{array}{ccc} U_{C}\left(t\right) & = & A_{1}e^{p_{{}_{1}}t}+A_{2}e^{p_{{}_{2}}t}+\frac{r_{a}}{r_{a}+R}\;U_{S}\left(0^{+}\right),\\ p_{1,2} & = & \frac{-RC_{p}r_{a}-L_{a}\pm \sqrt{{\left(RC_{p}r_{a}+L_{a}\right)}^{2}-4RC_{p}L_{a}\left(R+r_{a}\right)}}{2RC_{p}L_{a}}, \end{array}\;\right.$$

where $A_{1}$ and $A_{2}$ are constants, and they can be determined by the initial conditions. $U_{S}\left(0+\right)$ is the high voltage of the step input.

The step input is assumed to be zero until the inertial system is at the initial state. Consequently, a rising edge of the step input produces a response with an initial state of zero. The zero-initial condition of the differential function is

(6) $$\left\{\begin{array}{ccc} U_{C}\left(0^{+}\right) & = & 0,\\ i_{L}\left(0^{+}\right) & = & 0. \end{array}\;\right.$$

Substituting the first initial condition of Equation (6) into Equation (5), a constraint of $A_{1}$ and $A_{2}$ can be represented as follows:

(7) $$U_{C}\left(0^{+}\right)=A_{1}+A_{2}+\frac{r_{a}}{r_{a}+R}\;U_{S}\left(0^{+}\right)=0.\;$$

According to Equation (6), when $t=0^{+}$, the first KVL equation of Equation (2) can be written as follows:

(8) $${\left.\frac{dU_{C}}{dt}\right|}_{t=0^{+}}=\frac{U_{S}\left(0^{+}\right)-U_{C}\left(0^{+}\right)-Ri_{L}\left(0^{+}\right)}{RC_{p}}=\frac{U_{S}\left(0^{+}\right)}{RC_{p}}.\;$$

According to Equation (8) and differentiating Equation (5), another constraint of $A_{1}$ and $A_{2}$ can be represented as follows:

(9) $${\left.\frac{dU_{C}}{dt}\right|}_{t=0^{+}}=p_{{}_{1}}A_{1}+p_{{}_{2}}A_{2}=\frac{U_{S}\left(0^{+}\right)}{RC_{p}}.\;$$

Combining Equations (7) and (9), $A_{1}$ and $A_{2}$ can be solved. Thus, the solution of this inertial system is

(10) $$\left\{\begin{array}{ccc} U_{C}\left(t\right) & = & A_{1}e^{p_{{}_{1}}t}+A_{2}e^{p_{{}_{2}}t}+\frac{r_{a}}{r_{a}+R}\;U_{S}\left(0^{+}\right),\\ A_{1} & = & \frac{\frac{1}{RC_{p}}+p_{{}_{2}}\frac{r_{a}}{R+r_{a}}}{p_{{}_{1}}-p_{{}_{2}}}U_{S}\left(0^{+}\right),\\ A_{2} & = & \frac{\frac{1}{RC_{p}}+p_{{}_{1}}\frac{r_{a}}{R+r_{a}}}{p_{{}_{2}}-p_{{}_{1}}}U_{S}\left(0^{+}\right),\\ p_{{}_{1}} & = & \frac{-RC_{p}r_{a}-L_{a}+\sqrt{{\left(RC_{p}r_{a}+L_{a}\right)}^{2}-4RC_{p}L_{a}\left(R+r_{a}\right)}}{2RC_{p}L_{a}},\\ p_{{}_{2}} & = & \frac{-RC_{p}r_{a}-L_{a}-\sqrt{{\left(RC_{p}r_{a}+L_{a}\right)}^{2}-4RC_{p}L_{a}\left(R+r_{a}\right)}}{2RC_{p}L_{a}}, \end{array}\;\right.$$

where $U_{S}\left(0+\right)$ is the high voltage of the step input.

The measurement system of IPS takes samples of the response waveform of the inertial system and calculates the distance between the sensing core and the target. The voltage waveform on the measurement node must be overdamped, and the voltage waveform must be non-oscillation. Therefore, the characteristic equation presents two distinct real roots. The parameters of the inertial system satisfy the following equation:

(11) $${\left(RC_{p}r_{a}+L_{a}\right)}^{2}-4RC_{p}L_{a}\left(R+r_{a}\right)>0.\;$$

The equivalent distributed capacitance of the cable is the key element to decide whether the condition above is true. The value of $C_{p}$ determines whether the inertial system is in the overdamped case. Hence, the equation above is formalized as follows:

(12) $$f\left(C_{p}\right)={\left(Rr_{a}\right)}^{2}C_{p}^{2}-2RL_{a}\left(r_{a}+2R\right)C_{p}+L_{a}^{2}>0.\;$$

**Figure 3:**
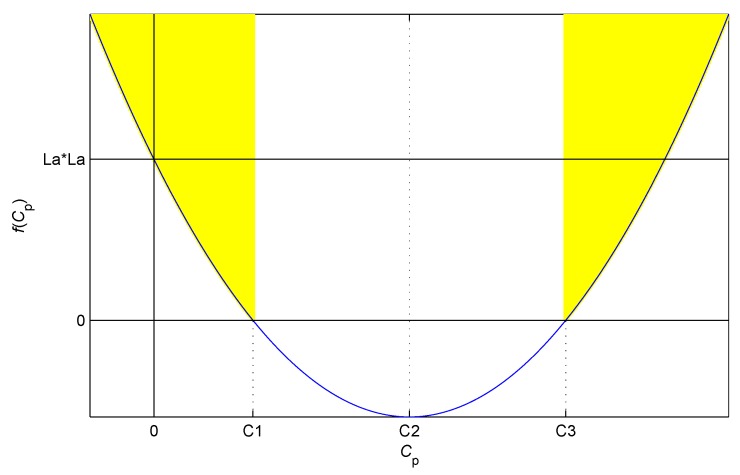
Damped cases of the system determined by the value of $f(C_{p})$.

The parameters in Equation (12) are physical quantities, and they belong to the positive real domain. Thus, the curve of $f(C_{p})$ is a parabola opening upward passing through $\left(0,La^{2}\right)$, and its symmetry axis is positive as shown in Figure 3.

When $C_{p}$ is less than $C_{1}$ or greater than $C_{3}$, the value of $f(C_{p})$ is positive; therefore, the inertial system is in the overdamped case. When $C_{p}$ equals $C_{1}$ or $C_{3}$, the value of $f(C_{p})$ is zero; therefore, the inertial system is in the critically damped case. When $C_{p}$ is greater than $C_{1}$ and less than $C_{3}$, the value of $f(C_{p})$ is negative; therefore, the inertial system is in the underdamped case. The sampling and calculation methods in this study require the inertial system satisfying the overdamped case.

Several typical parameters of the sensing core are selected as follows: *R* is 300 Ω, $L_{a}$ is 5.046 mH (approximately 3.5 mm distance, 100 m cable), and $r_{a}$ is 21.67 Ω (approximately 20 ${}^{\circ }$C, 100 m cable). $C_{1}$ is 13.53 nF, $C_{2}$ is 22.27 μF, and $C_{3}$ is 44.52 μF. The equivalent parallel capacitor of 100 m cable is 7.24 nF. The cable of non-integrative IPS is generally far less than 100 m; thus, the inertial system is reliably in the overdamped case.

**Figure 4:**
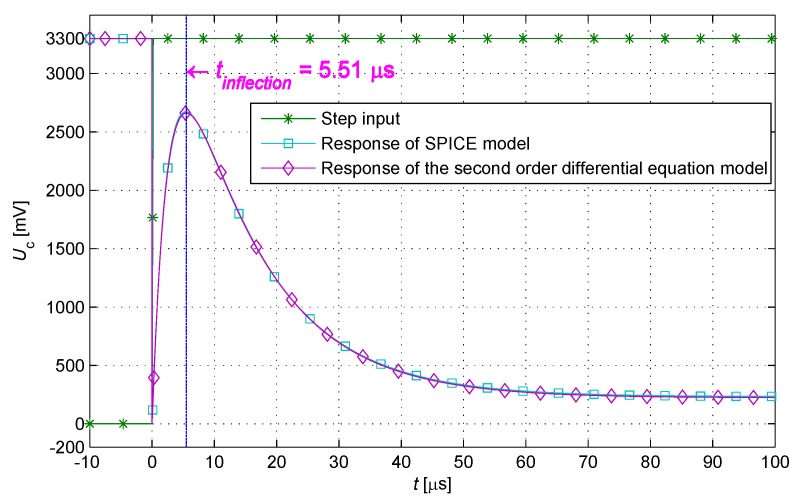
Calculation and simulation response waveforms of the inertial system.

In accordance with the parameters above, a calculation response waveform of the step input is calculated on the basis of the solution of the inertial system. A simulation response waveform is obtained using the Simulation Program with Integrated Circuit Emphasis software of Cadence (v17.2-2016, San Jose, CA, USA). The simulation and calculation responses are quite similar as shown in Figure 4.

## 3. Look-up Table Algorithm

The engineering parameters of IPS (including the cable length) satisfy Equation (12), that is, the corresponding inertial system is in the overdamped case. As constrained by the characteristic of the second-order differential equation, one and only one inflection point exists in the response waveform of the step input when t>0 (Figure 4). The specific moment at the occurrence of the inflection point ($t_{\mathrm{inflection}}$) can be calculated by solving Equation (13) as follows:

(13) $$U_{C}^{\prime }\left(t\right)=p_{{}_{1}}A_{1}e^{p_{{}_{1}}t}+p_{{}_{2}}A_{2}e^{p_{{}_{2}}t}=0.\;$$

At specific moments $t_{1}$ and $t_{2}$ after the rising edge of the step input, ADC is controlled to conduct two times of sampling. The samples $U_{1}$ at $t_{1}$ and $U_{2}$ at $t_{2}$ can be represented in Equation (14) as follows:

(14) $$\left\{\begin{array}{ccc} U_{1} & = & A_{1}e^{p_{{}_{1}}t_{1}}+A_{2}e^{p_{{}_{2}}t_{1}}+\frac{r_{a}}{r_{a}+R}\;U_{S}\left(0^{+}\right),\\ U_{2} & = & A_{1}e^{p_{{}_{1}}t_{2}}+A_{2}e^{p_{{}_{2}}t_{2}}+\frac{r_{a}}{r_{a}+R}\;U_{S}\left(0^{+}\right),\\ A_{1} & = & \frac{\frac{1}{RC_{p}}+p_{{}_{2}}\frac{r_{a}}{R+r_{a}}}{p_{{}_{1}}-p_{{}_{2}}}\;U_{S}\left(0^{+}\right),\\ A_{2} & = & \frac{\frac{1}{RC_{p}}+p_{{}_{1}}\frac{r_{a}}{R+r_{a}}}{p_{{}_{2}}-p_{{}_{1}}}\;U_{S}\left(0^{+}\right),\\ p_{{}_{1}} & = & \frac{-RC_{p}r_{a}-L_{a}+\sqrt{{\left(RC_{p}r_{a}+L_{a}\right)}^{2}-4RC_{p}L_{a}\left(R+r_{a}\right)}}{2RC_{p}L_{a}},\\ p_{{}_{2}} & = & \frac{-RC_{p}r_{a}-L_{a}-\sqrt{{\left(RC_{p}r_{a}+L_{a}\right)}^{2}-4RC_{p}L_{a}\left(R+r_{a}\right)}}{2RC_{p}L_{a}}. \end{array}\;\right.$$

In Equation (14), sampling moments $t_{1}$ and $t_{2}$ are constants; the high voltage of the step input $U_{S}\left(0^{+}\right)$ is constant; the current-limiting resistance *R* is constant; the sample values $U_{1}$ and $U_{2}$ are known quantities; the equivalent distributed capacitance of the cable $C_{p}$ is fixed and can be measured and recognized as constant. $L_{a}$ is constituted by $L_{w}$ and $L_{i}$: $L_{w}$ is the equivalent distributed inductance of the cable, which is fixed and can be measured; $L_{i}$ is the inductance component of the sensing coil, which varies with the distance between the sensing core and the target. $r_{a}$ is constituted by $r_{w}$ and $r_{i}$: $r_{w}$ is the internal resistance of the cable, which is fixed and can be measured; $r_{i}$ is the resistance component of the sensing coil, which changes with the ambient temperature. Then, two independent variables $L_{a}$ and $r_{a}$ are included in Equation (14). Given the two independent constraints in Equation (14), solutions of Equation (14) are obtained. When the inertial system is in the overdamped case and $t_{\mathrm{inflection}}<t_{1}<t_{2}$, the relevant portion of the response is continuous and monotonic. Thus, Equation (14) has one and only one solution. In other words, given the sample values $\left(U_{1},U_{2}\right)$ and the constants of parameters, $L_{a}$ and $r_{a}$ corresponding with the physical model can be solved.

A numerical solution of Equation (14) that satisfies an engineering accuracy requirement can be obtained. Subjecting to (s.t.) the given conditions, the values of $L_{a}$ and $r_{a}$ that minimize (min) the equation below are the numerical solution of Equation (14):

(15) $$\begin{array}{ccc} \min & : & {\left[U_{1}-{\left.U_{C}\left(L_{a},r_{a},t\right)\right|}_{t=t_{1}}\right]}^{2}+{\left[U_{2}-{\left.U_{C}\left(L_{a},r_{a},t\right)\right|}_{t=t_{2}}\right]}^{2},\\ s.t.1 & : & L_{a}>0,r_{a}>0,\\ s.t.2 & : & 0<t_{\mathrm{inflection}}<t_{1}<t_{2},\\ s.t.3 & : & {\left(RC_{p}r_{a}+L_{a}\right)}^{2}-4RC_{p}L_{a}\left(R+r_{a}\right)>0. \end{array}\;$$

A look-up table is established via calibration and offline calculation. Using the sample values $\left(U_{1},U_{2}\right)$, the distance between the sensing core and the target can be obtained by searching the look-up table. The reduction of complexity in system online computation can avoid the use of MCU and DSP.

### 3.1. Two-Dimensional Look-Up Table

Recording all the $\left(U_{1},U_{2}\right)$ combinations into a two-dimensional look-up table is an efficient and intuitive method. Using the sample values, the distance between the sensing core and the target can be obtained by searching the look-up table. In accordance with Equation (15), the values of inductance component $L_{i}$ corresponding to the $\left(U_{1},U_{2}\right)$ combinations in the look-up table are calculated via offline calculation. The two-dimensional look-up table regarding the sample values ($\left(U_{1},U_{2}\right)$ and the distance is established by the following procedures: using a precise electronic controlled displacement platform, adjusting the distance between the sensing core and the target by steps, measuring the value of inductance component $L_{i}$ via precision inductance meter, and establishing the two-dimensional look-up table indicating the relationship between $L_{i}$ and the distance.

**Figure 5:**
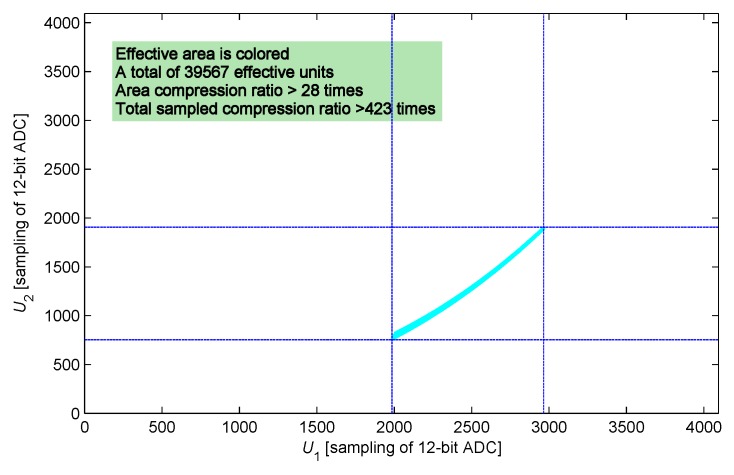
Definition domain of the two-dimensional look-up table.

The two-dimensional look-up table has a very large size originally. For example, the size of a complete two-dimensional look-up table has $2^{12}\times 2^{12}$ units when using a 12-bit ADC; this size indicates inapplicability. $L_{i}$ and $r_{i}$ are the parameters of the sensing core, and they belong to a positive real domain. Moreover, the definition ranges of value of $L_{i}$ changing with approaching status and value of $r_{i}$ changing with the ambient temperature are fixed. According to the constraints above, the size of the look-up table can be reduced dramatically by simply recording the effective data. Several typical IPS parameters are selected as follows: $L_{i}$ is 4.5–10.0 mH, $r_{i}$ is 8–20 Ω, $L_{w}$ is 4.57 μH (10 m cable), $r_{w}$ is 847 mΩ, and $C_{p}$ is 987 pF. Following the current-limiting condition, current-limiting resistance *R* takes a value of 300 Ω. At the moment, the response waveform reduces to approximately 60% and 30%, and $t_{1}$ and $t_{2}$ are calculated as 11.4 and 26.8 μs. A definition domain of the two-dimensional look-up table is established on the basis of the conditions above and recorded into the shadow (Figure 5). A total of 39,567 effective units are obtained in the look-up table after condensing.

### 3.2. Dimension Reduction of the Look-Up Table

**Figure 6:**
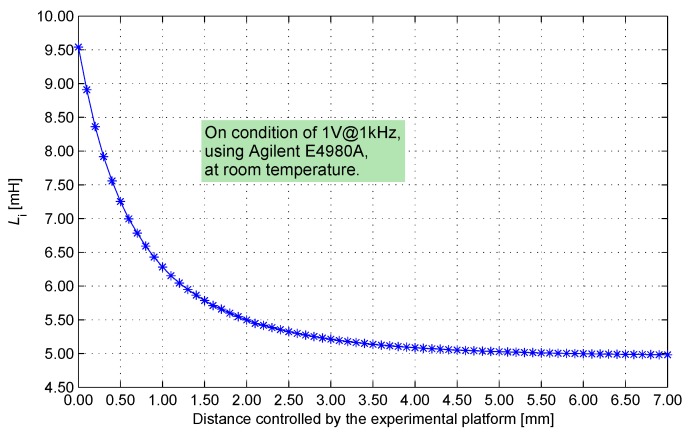
Calibration of distance vs. *L*.

The two-dimensional look-up table method can be effectively applied to the IPS system but exhibits the following disadvantages:

1. The look-up table is in a large scale, and the processing logic accesses the look-up table from the external non-volatile memory. These conditions negatively affect the stability and miniaturization of the IPS system.
2. The production tolerance of the sensing coil is usually approximately 3%, which is significantly larger than the system measurement precision. In the manufacturing process, the parameter of every product should be measured accurately, and a customized look-up table should be developed via complicated offline calculation. These conditions negatively affect mass production.
3. As the distance between the sensing core and the target decreases, the inductance component of the sensing coil expands rapidly in a nonlinear form (Figure 6). At the same distance interval, short distance means significant changes in the inductance component. However, the look-up table is recorded in the format of a uniform interval of inductance component. Accordingly, the distribution of measurement revolution becomes non-uniform. The measurement revolution is extremely high when the distance is small. The look-up table contains much redundant information and causes storage resource waste; the revolution decreases when the distance is large, thereby negatively affecting the detecting range expansion of the IPS system.

The method is limited mainly by its synchronous dependency on the complex offline computation procedure and the calibration procedure. However, the two procedures can be appropriately combined only when the relationship between the distance and the sample values $\left(U_{1},U_{2}\right)$ is calibrated at every temperature. Obviously, the relationships between the distance and the sample values $\left(U_{1},U_{2}\right)$ at dozens of constant temperatures cannot be calibrated. This study focuses on the nonlinear relationship between the response waveforms and the values of resistance component $r_{i}$, uses the linear approximation method, and proposes a new method. The proposed method reduces the times of constant temperature calibration significantly and decreases the look-up table dimensions.

When the response of the sensing coil is close to a steady state, the voltage on the measurement node is close to a constant and does not correlate with the inductance component $L_{i}$. Setting $t_{2}$ as infinity, Equation (14) can be simplified as follows:

(16) $$\left\{\begin{array}{ccc} U_{1} & = & A_{1}e^{p_{{}_{1}}t_{1}}+A_{2}e^{p_{{}_{2}}t_{1}}+\frac{r_{a}}{r_{a}+R}\;U_{S}\left(0^{+}\right),\\ U_{2} & = & \frac{r_{a}}{r_{a}+R}\;U_{S}\left(0^{+}\right). \end{array}\;\right.$$

An analytic expression of $r_{a}$ is obtained by the constraint of $U_{2}$ in Equation (16). Substituting the expression into the constraint of $U_{1}$ separates the inductance component.

Two samplings are taken at $t_{1}$ and $t_{2}$ by ADC. Substituting the sample values into Equation (16) solves the inductance component of the sensing coil. A look-up table method can simplify the calculation. Considering that $L_{a}$ and $r_{a}$ are separated, the format of the two-dimensional look-up table changes into the relationship between $L_{a}$ and $U_{1}$ for each of the specific values of $r_{a}$. In Table 1, the rows from left to right show the growth direction of the free variable $r_{a}$; the columns from top to bottom show the growth direction of the free variable $L_{a}$. Each specific sampling value of $U_{2}$ corresponding to each of $r_{a}$ indicates the operation temperature of IPS. Furthermore, a cluster of sampling values of $U_{1}$ exist and indicate the distances between the sensing core and the target at the specific temperature (Table 1).

**Figure 7:**
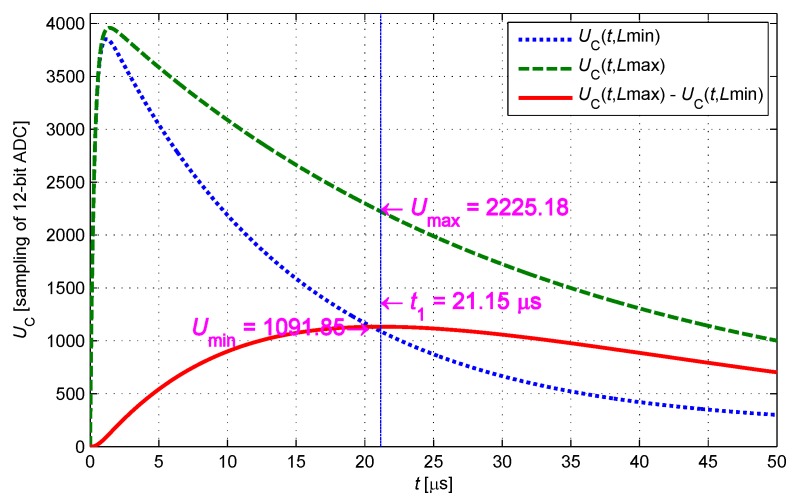
Optimization of $t_{1}$.

The constant temperature calibrating procedure is repeated for each *r* to completely establish the data in Table 1. The times of constant temperature calibration can be reduced by taking advantage of the linear characteristic of the data distribution. In the numerical analysis, several typical IPS parameters are selected as follows: *L* is 4.5–10.0 mH, and *r* is 8–20 Ω. Following the current-limiting condition, current-limiting resistance *R* takes a value of 300 Ω. $t_{2}$ is obtained as 250 μs using the constraint in Equation (17), the response of the sensing coil is close to a steady state at this moment, and the variance with terminal voltage is less than one of the least significant bit (LSB) of ADC. The sample value at $t_{2}$ represents the terminal voltage, and the sampling at $t_{1}$ is the only effective sample during the response curve changing process. $t_{1}$ is regarded as the moment when the response curve changes most significantly. As shown in Figure 7, $t_{1}$ is obtained as 21.15 μs using the constraint in Equation (18), and when $r_{i}$ is 13.2 Ω (at room temperature) and the cable length is 10:

(17) $$\begin{array}{ccc} U_{{}_{C}}\left(t_{2}\right)-U_{{}_{C}}\left(+\infty \right) & = & A_{1}e^{p_{{}_{1}}t_{1}}+A_{2}e^{p_{{}_{2}}t_{1}}<\frac{U_{S}\left(0^{+}\right)}{2^{n}-1}, \end{array}$$

(18) $$\begin{array}{c} {\left.\frac{d({\left.U_{{}_{C}}\left(t\right)\right|}_{L_{{}_{a}}=10+0.00457\mathrm{mH}}-{\left.U_{{}_{C}}\left(t\right)\right|}_{L_{{}_{a}}=4.5+0.00457\mathrm{mH}})}{dt}\right|}_{R=300\Omega ,r_{{}_{a}}=13.2+0.847\Omega ,C_{{}_{p}}=987\mathrm{pF}}=0. \end{array}$$

**Figure 8:**
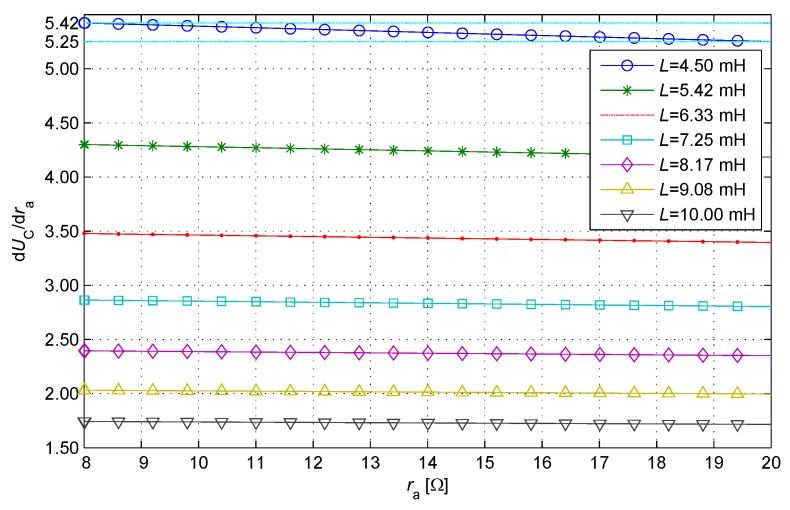
Non-linear relationship between the sample value and the resistance component.

The parameters above are used to analyze the nonlinear relationship between the sample value and the resistance component, $t_{1}$ is regarded as a constant, $L_{a}$ and $r_{a}$ are regarded as variables. Consequently, an analytic expression of the partial derivative of $U_{C}\left(L_{a},r_{a}\right)$ with respect to $r_{a}$ is obtained. The nonlinear relationship between the sample value and the resistance component is drawn using the analytic expression taking uniform sampling in the inductance component domain (Figure 8).

In Figure 8, the curve of $\partial U_{c}\left(L_{a},r_{a}\right)/\partial r_{a}$ is close to a horizontal line (constant), indicating that the curve of $U_{c}\left(r_{a}\right)$ is close to a straight line. When $L_{a}$ is the minimum (the distance is the maximum), the nonlinear degree of the curve is the largest. In particular, the accumulated deviation of the curve is approximately 0.17 times of the LSB of ADC. This condition means the relationship between the sample value and the resistance component is close to a linear relationship for the typical parameters of IPSs. Thus, the error of linear interpolation can be neglected for these IPSs with 12-bit ADCs.

### 3.3. Implementation of the Dimension-Reduced Method

**Figure 9:**
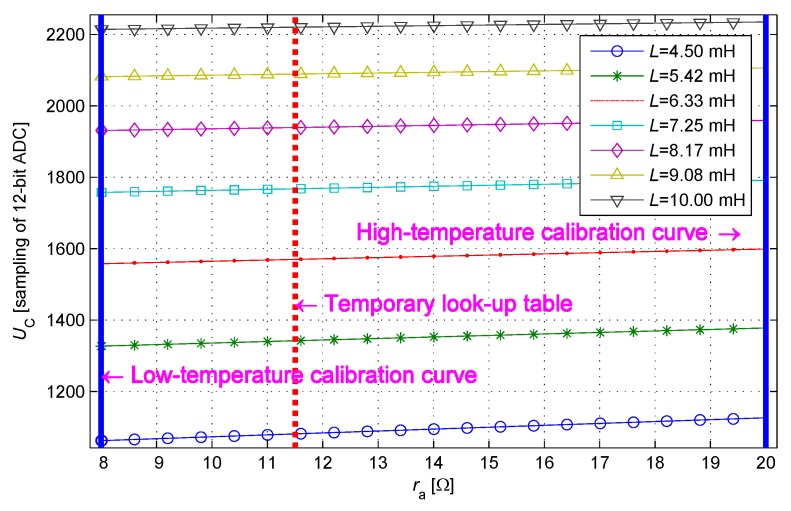
Cluster of $U_{C}\left(L_{a},r_{a}\right)$ curves.

Calibrating the relationship between the distance and the sample value $U_{1}$ at two constant temperatures obtains the high- and low-temperature calibration curves, and the corresponding $U_{2L}$ and $U_{2H}$ (Figure 9). $U_{2L}$ and $U_{2H}$ correspond to the resistance component $r_{i}$ at two calibration temperatures. A measurement procedure of IPS obtains the sample values $\left(U_{1},U_{2}\right)$. At the ambient temperature testified by $U_{2}$, the relationship between the distance and the sample value $U_{1}$ is close to a linear relationship. The proportional relationship between the sample value $U_{2}$ and $\left(U_{2L},U_{2H}\right)$ determines the horizontal position of the interpolation curve in Figure 9. After obtaining the proportion and the high- and low-temperature calibration curves, the temporary look-up table can be established. Searching the location of the sample value $U_{1}$ in the temporary look-up table determines the distance between the sensing core and the target. Thus, the two-dimensional look-up table is simplified into two one-dimensional look-up tables.

As the resistance component $r_{i}$ is proportional to the ambient temperature of IPS, the high-temperature calibrating procedure does not need to be processed at extremely high temperatures. The high-temperature environment can be simulated at room temperature by applying a resistance into the charging circuit in series. This resistance does not have to be accurate but should be stable. Usually, a metal film resistor with 1% tolerance and in the range of 5 Ω to 10 Ω is chosen. As no negative physical resistance exists, series resistance cannot be used to simulate a low-temperature environment. The sample environment needs not be set into extreme status in the linear interpolation, and the low-temperature calibrating procedure needs not be processed at extremely low temperature. Instead, the calibrating procedure can be processed at room temperature. Calibrating at ambient temperature simplifies the production procedure.

IPS is driven by a precise electronically controlled displacement platform to move at the step of 0.1 mm in the range of 0–7 mm. A total of 71 sets of calibrating data are found in a set of sample values $\left(U_{1},U_{2}\right)$ from IPS on each calibrating point. All the sample values $U_{2}$ are nearly equal; thus, the mean value of $U_{2}$ can be recorded. The procedure above is implemented at two calibration temperatures, and the high- and low-temperature calibration look-up tables are established completely as shown in Table 2.

Combining the calibrating data, a linear interpolation of actual sampling $U_{2S}$ of IPS is processed by

(19) $$\begin{array}{ccc} U_{1T\_i} & = & \left(U_{1H\_i}-U_{1L\_i}\right)\frac{U_{2S}-U_{2L}}{U_{2H}-U_{2L}}+U_{1L\_i}\;,\\ i & = & 0,1,2,\ldots ,70\;, \end{array}\;,$$

and the temporary look-up table at the ambient temperature is obtained. These calculations are made online and can be simplified by the following deduction:

(20) $$\begin{array}{ccc} U_{1T\_i} & = & U_{2S}M_{i}+N_{i}\;,\\ M_{i} & = & \frac{U_{1H\_i}-U_{1L\_i}}{U_{2H}-U_{2L}}\;,\\ N_{i} & = & \frac{U_{2H}U_{1L\_i}-U_{2L}U_{1H\_i}}{U_{2H}-U_{2L}}\;,\\ i & = & 0,1,2,\ldots ,70\;, \end{array}\;,$$

where operators $M_{i}$ and $N_{i}$ are constants calculated offline according to high- and low-temperature calibration look-up tables.

Combining the high- and low-temperature calibration look-up tables, calibration operators $M_{i}$ and $N_{i}$ are obtained via offline calculation and recorded in the memory of IPS. The IPS working procedures are as follows: applying pulse excitation on the sensing core; controlling ADC to take sample twice, and obtaining sample values $\left(U_{1S},U_{2S}\right)$; using Equation (20) and taking 71 times of multiply–add operations with $U_{2S}$ and the calibration operators, the temporary look-up table is established; comparing the sample value $U_{1S}$ with the temporary look-up table (Table 3), the result shows that the integer part of the distance is *x* × 0.1 mm.

The decimal part of the distance is estimated, and the modified distance is obtained by further interpolation calculation using the adjacent data and Equation (21):

(21) $$\begin{array}{ccc} D & = & 0.1\left(x-\frac{U_{1S}-U_{1T\_x}}{U_{1T\_(x-1)}-U_{1T\_x}}\right)\;\mathrm{mm}\;,\\ i & = & 0,1,2,\ldots ,70\;. \end{array}$$

The linear interpolation significantly reduces the dimension of the look-up table and the indexing consumption. The size of the dimension-reduced look-up table is reduced to 144 units. The processing logic circuit accesses the calibration data from the inner random-access memory, which is loaded from the external read-only memory chips at the initial stage. As the accessing frequency of the external bus dramatically decreases, the system stability improves. The complete parameter information at the dimensions of the ambient temperature and the distance is established via calibration and interpolation. This procedure avoids precise measurement on IPS sensing core and cable, and completely offsets inconsistency of sensing core production. The dimension-reduced look-up table is distributed at the calibration distance interval. The measurement precision and the range of the key measurement areas can be flexibly selected by adjusting the distribution of calibration interval.

## 4. Discussion

### 4.1. Precision of Interpolation

A linear approximation method is used to substitute the two-dimensional look-up tables with two one-dimensional look-up tables for calculation. This procedure is necessary to evaluate whether the interpolation error of the method can satisfy engineering measurement accuracy.

**Figure 10:**
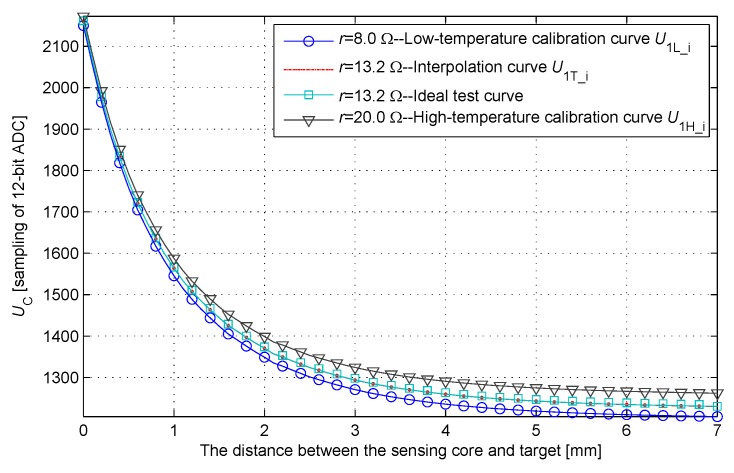
Cluster of $U_{C}$ (distance) curves.

IPS is driven by precise electronically controlled displacement platform to move at the interval of 0.1 mm in the range of 0–7 mm. A precision inductance meter is used to measure the value of inductance component $L_{i}$ synchronously, and the relationship between $L_{i}$ and the distance is established. In accordance with Equation (10), the corresponding relationship between the sample value and the distance at $t_{1}$ is established when resistance component $r_{i}$ is 8.0 and 20.0 Ω. The high- and low-temperature calibration curves are drawn as shown in Figure 10. The same method is applied to calculate an ideal test curve for error analysis given the resistance component $r_{i}$ of 13.2 Ω. The interpolation curve at the same ambient temperature is calculated using Equation (20). The interpolation curve is very close to the ideal test curve.

**Figure 11:**
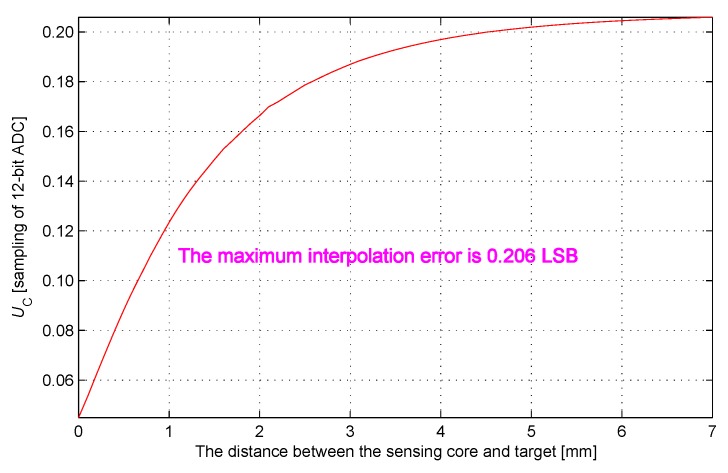
Distribution of interpolation error.

The distribution of interpolation error is obtained by calculating the difference between the interpolation curve and the ideal test curve, and is shown in Figure 11. The maximum interpolation error is 0.206 LSB, which testifies that the interpolation error can satisfy the IPS measurement requirement.

This analysis of interpolation error only applies to kinds of typical IPS parameters. The accuracy of linear interpolation may not be able to meet the requirements if the parameter changes significantly or the measurement distance becomes large. Highly precise interpolation methods can be performed to expand the adaptability of this method by establishing additional temperature calibration curves. The main examples of these interpolation methods are the quadratic spline interpolation and the cubic spline interpolation, which can indefinitely improve the accuracy of linear interpolation. The clearance fit of the IPS metal shell and the sensing coil is slightly affected by the ambient temperature, which constrains infinite improvement of IPS measurement accuracy. Linear and quadratic spline interpolations usually meet most of the application requirements.

### 4.2. Built-in Self-Test

**Figure 12:**
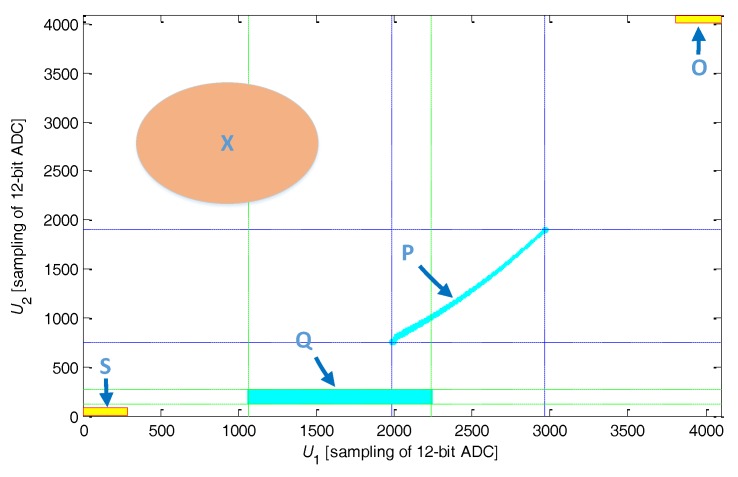
Built-in self-test vector diagram.

BIST is an important function of IPS, especially for highly reliable fields. This function can be easily established and realized on the basis of the two-dimensional look-up table. The obtained BIST function is implemented not only in the system initialization process but also in the system working process. This feature is one of the key advantages of these methods. As shown in Figure 12, the shadow area **P** is the definition domain of the two-dimensional look-up table. If the sample value $\left(U_{1},U_{2}\right)$ falls into area **P**, then the system is in the normal condition. Otherwise, the system may have a fault or fault potential. For example, if the sample value falls into area **S**, then a short-circuit in the sensing coil or a failure of ADC occurs; if the sample value falls into area **O**, then a broken-circuit in the sensing core or a failure in the driving circuit occurs. Area **X** testifying other failure modes can be expanded by taking further statistics and analysis on the typical IPS failure modes. The procedure is implemented at each sampling period; thus, the system failure status can be determined in real time. As for the dimension-reduced look-up table, the definition domain of sample value $\left(U_{1},U_{2}\right)$ is mapped from area **P** to area **Q** when $t_{2}$ becomes large. Remapping of sample domain is made without compromising the BIST procedure.

### 4.3. Comparison of the Principal Character and the Original Method

The dimension-reduced measurement method is introduced into the original two-dimensional look-up table method to realize the IPS system. This study analyzes the advantages and disadvantages of the original method. Aiming at overcoming the disadvantages of the two-dimensional look-up table method, a dimension-reduced measurement method is proposed. By setting the second sampling moment ($t_{2}$) as “infinity”, the inductance and resistance components of the inertial system are separated. The second sample value ($U_{2}$) represents the ambient temperature, and the first sample value ($U_{1}$) represents the distance between the sensing core and the target at specific ambient temperature. An approximate linear relationship is found between the sample value and the resistance component. The high- and low-temperature calibration look-up tables are established by the calibration procedure. In the final stage, a linear interpolation method is used to calculate the distance between the sensing core and the target at operating temperature by searching the calibration look-up tables.

### 4.4. Optimization of the Driving Waveform

If a driving waveform can generate a monotonic response in the measurement window, then the solution for this inertial system can be found. This kind of driving waveforms can take reference of the method introduced in this study. However, the step input shows its unique advantages, which make it very suitable for the dimension-reduced measurement method.

Using sinusoidal input shows better electromagnetic compatibility than using the step input. As for the dimension-reduced measurement method, the sinusoidal input can cause a significant eddy-current effect, and the associative real part resistance cannot be directly separated from the resistance component of the IPS core. Using the step input, the eddy-current effect decreases rapidly after the rising edge of the step input; accordingly, the sample value at sampling moments $t_{1}$ (21.5 μs) is slightly affected. In addition, this slight effect can be constrained during the calibration procedure. At the sampling moments $t_{2}$ (250 μs), the eddy-current effect can be neglected nearly completely, and the measurement of the resistance component is unaffected.

The practicability value of this dimension-reduced measurement method mainly reflects the establishment of calibration look-up tables at only two simulated calibration temperatures under room temperature. This depends on an approximate linear relationship between the response of the sensing core and the ambient temperature; this relationship is restrained by the solution of the related inertial system. For the step input, the particular solution is constant, and an approximate linear relationship exists. When using other driving waveforms, the particular solution is variable. This phenomenon may lead to the disappearance of the approximate linear relationship and further loss of this advantage of the dimension-reduced measurement method.

The step input is a pretty good driving waveform for this dimension-reduced measurement method.

### 4.5. Drift of the Coil Parameter and Target

As mentioned in Section 1, the inductance component of the IPS core shows the distance between the sensing core and target. However, the permeability of the iron core in the IPS core changes with temperature. In this point, a temperature drift in the inductance component of the IPS occurs. This dimension-reduced measurement method cannot directly separate the inductance component change introduced by temperature drift. Therefore, serials of technics are used in IPS core production to reduce the temperature drift of permeability.

In a related experiment, an iron core made of Q235A (a common carbon steel) is assembled into an IPS coil. The inductance variance of this coil (away from the target) is 0.273 mH (319 PPM/${}^{\circ }$C) when the temperature ranges from −55 ${}^{\circ }$C to 70 ${}^{\circ }$C. From the data in Figure 6, this variance is equivalent to the inductance variance when the distance ranges from 7 mm to 2.8 mm. An iron core made of EJ79 (a kind of permalloy) with full stress relaxation and an annealing treatment is assembled into an IPS coil. The inductance variance of this coil (away from the target) is 0.005 mH (8 PPM/${}^{\circ }$C) when the temperature ranges from −55 ${}^{\circ }$C to 70 ${}^{\circ }$C. From the data in Figure 6, the variance is equivalent to inductance variance when the distance ranges from 7 mm to 6.7 mm. Thus, the optimization of material and technics can effectively reduce the temperature drift of the inductance component of the IPS core.

The final value of the zero-state response shows the working temperature of the system. The temperature drift model of the inductance component can be established by conducting further statistics analysis. A one-dimensional lookup table can be established, and the temperature drift of the inductance component can be compensated by $U_{2}$.

The temperature drift of the target permeability leads to a temperature drift of the skin-depth of the target. For materials, such as iron core, the target also requires full stress relaxation and annealing treatment.

The magnetization intensities of the materials of the iron core and target are set in very high levels. To a certain degree, it reduces the risk that the iron core and target are magnetized further by the driving waveform (less than 15 mA). However, concerning magnetization accumulation, a driving circuit with symmetrical structure is designed. It is expected that the risk will be eliminated by alternately imposing step inputs both in positive and negative loops on the coil.

## 5. Results

**Figure 13:**
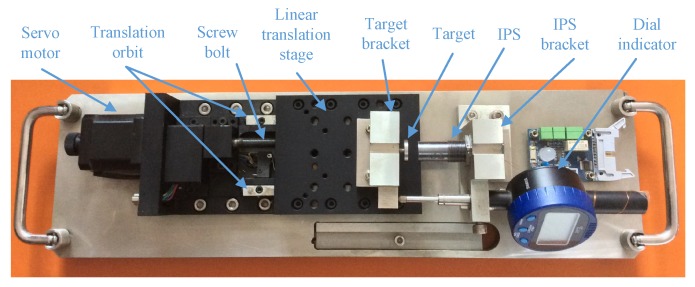
Precise electronically controlled displacement platform.

A precise electronically controlled displacement platform has been developed for the calibration and testing procedures. As shown in Figure 13, the IPS (or the sensing core section of the non-integrative IPS) is installed on the IPS bracket fixed on the displacement platform. The target is fitted on the linear translation stage. The servo motor drives the linear translation stage move at the axial directions of the translation orbit through the screw bolt. The dial indicator detects the distance between the IPS and the target, and provides the data to the controller of the displacement platform. The controller of the displacement platform automatically controls the distance between the IPS and the target by driving the servo motor, and performs the calibration and testing procedure. The re-orientation accuracy of the displacement platform is ±20 μm, which is ensured by the related metrology accreditation.

A batch of engineering prototypes are developed and used to test the proposed dimension-reduced measurement method. As mentioned above, the dimension reduction significantly reduces the look-up table size, thereby enabling the improvement of IPS measurement precision by increasing the resolution bits of ADC. Therefore, on the basis of the processing circuit structure of our previous study [12], a 14-bit ADC is used on the engineering prototype to enhance the sensitivity of IPS.

**Figure 14:**
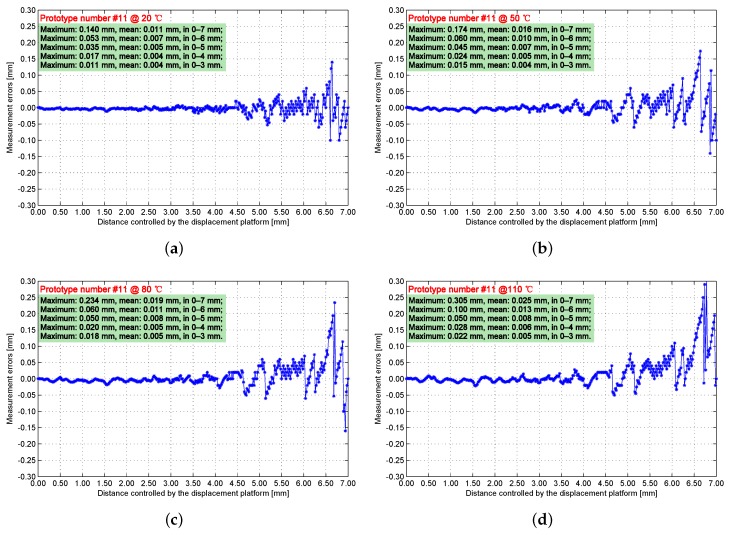
Measurement results of prototype #11 when ambient temperatures are (**a**) 20 ${}^{\circ }$C; (**b**) 50 ${}^{\circ }$C; (**c**) 80 ${}^{\circ }$C; and (**d**) 110 ${}^{\circ }$C.

Engineering prototype #11 is calibrated and tested by connecting the sensing core and the processing circuit with a 5 m cable. The measurement errors of engineering prototype #11 are taken at 20, 50, 80, and 110 ${}^{\circ }$C (Figure 14). These experimental data indicate that, when the distance between the IPS and the target is short, the measurement error becomes insignificant. As the distance decreases, the inductance component of the sensing coil expands rapidly in a nonlinear form. Meanwhile, the maximum and mean errors increase as temperature increases. The reason is due to the temperature drift of the displacement platform and the magnetic core of the IPS.

**Figure 15:**
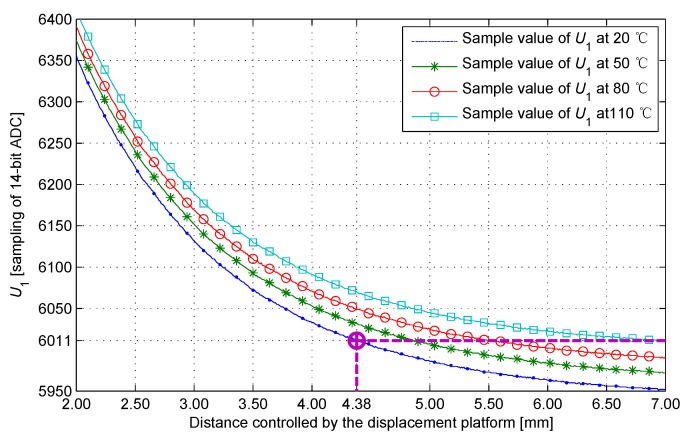
Sample value at different temperatures.

Figure 15 is labeled using the sample value $U_{1}$ from the four sets of experiments above. The sample value is significantly affected by temperature. If the ambient temperature effects are neglected, then the measurement errors of the IPS would increase significantly. For example, when the distance is 7 mm at 110 ${}^{\circ }$C, and using the calibration curves of 20 ${}^{\circ }$C to explain the current sample value, the distance is 4.38 mm, and the error is 2.62 mm. IPSs of this precision nearly have no practical value.

The only effective sample is taken during the response curve changing process, and the sample value $U_{1}$ is obtained. The sample value $U_{1}$ determines the position of the distance to be measured on the calibration curve. Meanwhile, the terminal voltage ($U_{2}$) of the response curve is determined. The sample value $U_{2}$ determines which calibration curve can be used for searching. The specific calibration curve is the temporary look-up table, which is calculated using the sample value $U_{2}$ and the calibration operators. This method separates the inductance and resistance components of the sensing core. Therefore, extracting the characteristic of the IPS response waveform, and separating the inductance and resistance components are the key points in improving the precision of the IPS system.

**Figure 16:**
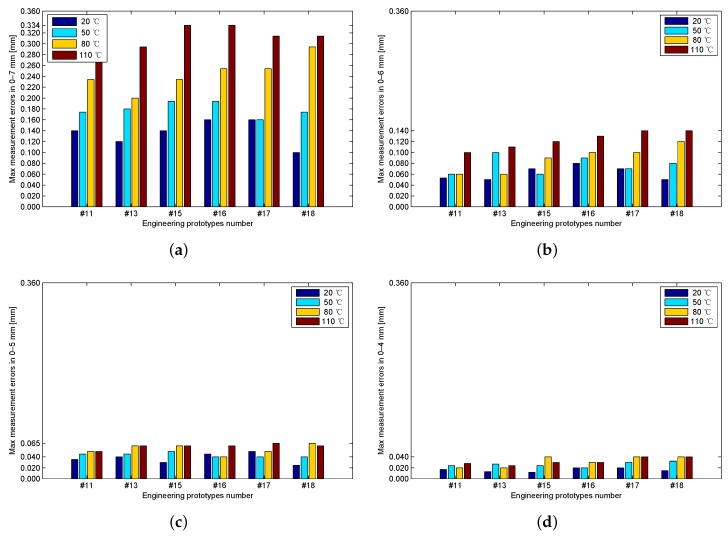
Maximum measurement errors of the six engineering prototypes when the measurement ranges are (**a**) 0–7 mm; (**b**) 0–6 mm; (**c**) 0–5 mm; and (**d**) 0–4 mm.

Six engineering prototypes are used to test the suitability of cable at different lengths and consistency of engineering prototypes. Engineering prototypes #11 and #13 are calibrated and tested using a 5 m cable connected between the sensing core and the processing circuit; engineering prototypes #15 and #16 are calibrated and tested using a 10 m cable; engineering prototypes #17 and #18 are calibrated and tested using a 20 m cable. As shown in Figure 16, at the same measurement range, no significant statistical differences are found in the maximum measurement errors by use of cables with different lengths; the maximum measurement error decreases with the measurement range. When the measurement ranges are 0–7, 0–6, 0–5, and 0–4 mm, the maximum measurement errors of the entire temperature ranges are 0.334, 0.140, 0.065, and 0.040 mm, respectively.

**Figure 17:**
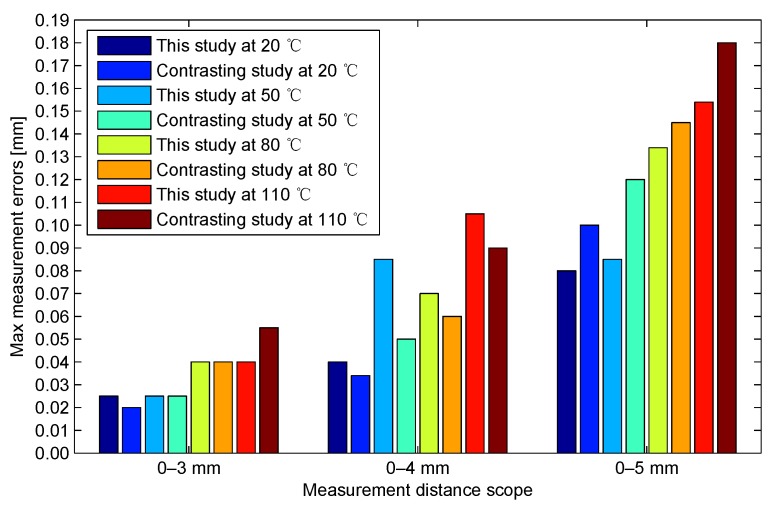
Comparison of measurement errors in the present study and those in the previous study.

Our previous study proposed a two-dimensional look-up table measurement method [12]. The testing prototypes in the previous study apply 12-bit ADCs, whereas the testing prototypes in the present study apply 14-bit ADCs. To enable comparison of the two methods in the same condition, the resolutions of the temperature calibration look-up tables and the sample values in the present study are reduced from 14-bit to 12-bit, by ignoring the two least significant bits. By recalculation of the current study, the bar graphs of the maximum measurement errors of the current study and the previous study are obtained at different typical ambient temperatures and measurement scopes (Figure 17). The distributions of the maximum measurement errors in the current study and those in the previous study are similar.

Notably, most of the maximum measurement errors in the current study are slightly less than those in the previous study. On the basis of the two-dimensional look-up table measurement method proposed in the previous study, the calculation process via the two-dimensional look-up table in the present study is replaced by linear interpolation via two one-dimensional look-up tables. Theoretically, the accuracy of the linear approximation method in the present study is slightly poorer than that of the straightforward calculation method in the previous study. The inconsistency is attributed to that the effective number of bits (ENOB) of the ADC used in the previous study is 10.5-bit, and the ENOB of the ADC used in the current study is 13.0-bit, which is better than that in the previous study even after being reduced to 12-bit. Therefore, if the interpolation error is controlled in a reasonable range, then the interpolation accords with the result solved by the model.

Some publicly released references with a few similarities to the present study are used for comparison. In particular, the chosen references have the same application background in the field of aviation and similar parameters, and they all avoid using process-control-based components. Table 4 shows the comparisons and highlights the advantages of the current study.

## 6. Conclusions

This study established a second-order inertial system for an analytical model of IPS and solved the constraint equation of the inertial system. The parameter constraint and parameter margin were introduced to ensure the second-order inertial system was in the overdamped case. After solving the constraint equation of the model, this method separated the inductance and resistance components of the IPS sensing core. Thus, the temperature drift was radically constrained.

This study considered the approximate linear relationship between the response of the sensing core and the ambient temperature, and proposed a dimension-reduced analog–digital mixed measurement method. This method complemented the disadvantages of the two-dimensional look-up table method in the following aspects:

- The size of the dimension-reduced look-up table was reduced from 39,567 units to 144 units. Thus, the memory consumption was significantly reduced, and the scale of the look-up table was controlled during the improvement of measurement precision. The reduction in look-up table size enabled the system access of the calibration data from an internal bus, thereby improving the system stability.
- The dimension reduction and linear interpolation enabled the establishment of calibration look-up tables via a displacement platform at only two simulated calibration temperatures under room temperature. The calibrating process completely offset the inconsistency of IPS sensing coil production, and avoided the complex offline computation and the precise measurement on the sensing core. This condition facilitated the scale production of IPS.
- The dimension-reduced look-up table was distributed at the calibration distance interval. Recording the calibration look-up table by use of the uniform growth of the distance improved the storage utilization efficiency and expanded the measurement precision.

To verify this dimension-reduced measurement method, a testing environment was constructed for evaluating the performance of the engineering prototypes. The experimental data showed that, when the measurement ranges were 0–6, 0–5, and 0–4 mm, the maximum measurement errors were 0.140, 0.065, and 0.040 mm, respectively, under the temperature ranging from 20 ${}^{\circ }$C to 110 ${}^{\circ }$C. This method was compared with that in the previous work based on the two-dimensional look-up table method under the same condition (by reducing the ADC resolution of this method from 14-bit to 12-bit). The distributions of the maximum measurement errors in the two studies were similar. Furthermore, the reduction in the look-up table dimensions of this method improved the sampling resolution from 12-bit to 14-bit with less memory consumption. Moreover, using the simplified calibration procedures, the measurement range was extended from 0–5 mm to 0–7 mm, and the measurement accuracy was improved over 0.1 mm (50% at 5 mm). Therefore, the proposed method not only achieved the expected capacity improvements but also improved the measurement precision and extended the measurement distance.

## Figures and Tables

**Table 1:** Two-dimensional look-up table with the separation of variables.

	$r_{\min}$	→	→	→	$r_{\max}$
	$U_{2\min}$	$U_{2\min}+1$	$U_{2\min}+2$	⋯	$U_{2\max}$
$L_{\min}$	$U_{1\_00}$	$U_{1\_10}$	$U_{1\_20}$	$U_{1\_i0}$	$U_{1\_y0}$
↓	$U_{1\_01}$	$U_{1\_11}$	$U_{1\_21}$	$U_{1\_i1}$	$U_{1\_y1}$
↓	$U_{1\_02}$	$U_{1\_12}$	$U_{1\_22}$	$U_{1\_i2}$	$U_{1\_y2}$
↓	⋯	⋯	⋯	⋯	⋯
$L_{\max}$	$U_{1\_0x}$	$U_{1\_1x}$	$U_{1\_2x}$	$U_{1\_ix}$	$U_{1\_yx}$

**Table 2:** High- and low-temperature calibration look-up tables.

	$U_{2L}$	$U_{2H}$
0.0mm	$U_{1L\_0}$	$U_{1H\_0}$
0.1mm	$U_{1L\_1}$	$U_{1H\_1}$
0.2mm	$U_{1L\_2}$	$U_{1H\_2}$
⋯	⋯	⋯
7.0mm	$U_{1L\_70}$	$U_{1H\_70}$

**Table 3:** Storage, calculation, and searching process of the look-up table.

	Calibration Operators $M_{i}$	Calibration Operators $N_{i}$	Temporary Look-Up Table $U_{1T\_i}$	Sample Value $U_{1S}$
0.0mm	$M_{0}$	$N_{0}$	$U_{1T\_0}$	Less than $U_{1T\_0}$
0.1mm	$M_{1}$	$N_{1}$	$U_{1T\_1}$	Less than $U_{1T\_1}$
0.2mm	$M_{2}$	$N_{2}$	$U_{1T\_2}$	Less than $U_{1T\_2}$
**⋯**	⋯	⋯	⋯	Less than ⋯
x×0.1mm	$M_{x}$	$N_{x}$	$U_{1T\_x}$	Greater than $U_{1T\_x}$
**⋯**	⋯	⋯	⋯	Greater than ⋯
7.0mm	$M_{70}$	$N_{70}$	$U_{1T\_70}$	Greater than $U_{1T\_70}$

**Table 4:** Comparison of the present study with related works.

	Present Study	Previous Study [12]	Application Specific Integrated Circuit [15]	Honeywell Model Number: ZS-00305 [7]
**Temperature drift reduction**	Self-adaptive compensation is unnecessary	Self-adaptive compensation is unnecessary	Customized thermal resistance compensation is required	Unknown
**Measurement method**	Analog–digital mixed	Analog–digital mixed	Analog	Analog
**Guaranteed actuation distance**	3.0 ± 0.04 mm or 4.0 ± 0.06 mm or 5.0 ± 0.10 mm or 6.0 ± 0.20 mm	4.5 ± 0.2 mm	3.28 ± 0.2 mm	4.0 ± 0.5 mm
**Quantitative method**	Yes	Yes	No	No
**Built-in self-test**	Yes, real time	Yes, real time	Yes, non-real time	Yes, non-real time
**Cable adaptability**	Yes	No	Yes	Unknown
**Adjusting of manufacturing deviation**	Direct calibration	Mixture of precision measurement and offline calculation	Direct calibration	Unknown
**Size of look-up table (units)**	144	49,725	None	None
**Input current (at 28VDC)**	6 mA	6 mA	4 mA	10 mA
**MCU or DSP**	Non-adoptive	Non-adoptive	Non-adoptive	Non-adoptive

## Abbreviations

The following abbreviations are used in this manuscript:

IPS	inductive proximity sensor
MTBF	high mean time between failure value
BIST	built-in self-test
TRAS	thrust reverser actuation systems
MCU	micro-controller unit
DSP	digital signal processor
ADC	analog-to-digital converter
KVL	Kirchhoff’s voltage law
LSB	least significant bit
ENOB	effective number of bits
